# Supplementary material for: SMARCD1 is an essential expression-restricted metastasis modifier
Source: Commun Biol. 2024 Oct 10;7:1299. doi: 10.1038/s42003-024-07018-3 (PMC11467182; doi:10.1038/s42003-024-07018-3)
Supplement: Supplementary file 1 — Supplementary Information [file 42003_2024_7018_MOESM1_ESM.pdf]

Supplemental Figures

Supplemental Figure 1

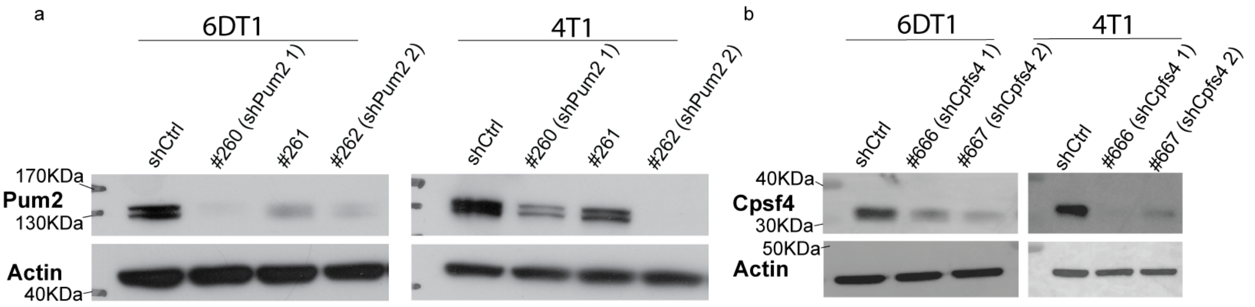

Figure S1. RNA-binding proteins Nanos1, Pum2, and Cpsf are metastasis modifiers in 6DT1 mouse mammary breast cancer cells.

a, western blot analysis of Pum2 protein levels in 6DT1 and 4T1 cells transduced with scramble shScr or three different shRNAs targeted at *Pum2* mRNA. b, western blot analysis of Cpsf4 protein levels in 6DT1 and 4T1 cells transduced with scramble shScr or three different shRNAs targeted at *Cpsf4* mRNA.

Supplemental Figure 2

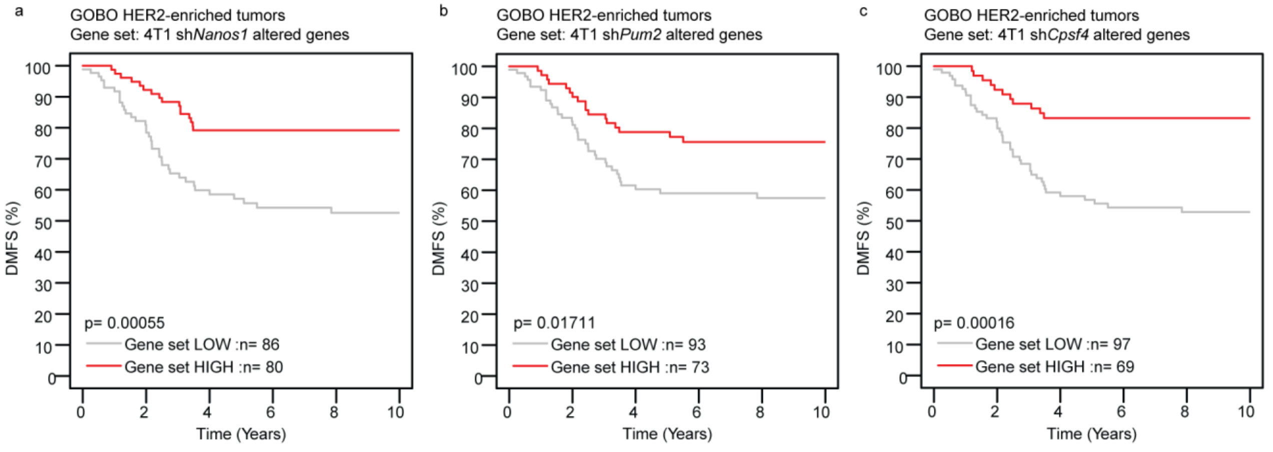

Figure S2. Differentially expressed genes from RNA-binding protein knockdown lines stratify ER+/HER2-enriched patient distant metastasis-free survival.

a-c, Kaplan-Meier analysis of distant metastasis-free survival (DMFS) for ER+/HER2-enriched patients stratified by differentially expressed gene lists from 4T1 sh*Nanos1* cells (a), sh*Pum2* cells (b), and sh*Cpsf4* cells (c) used as non-weighted signatures in the GOBO dataset.

Supplemental Figure 3

a

Gene	PRE UGUANAUA	NRE GUUGU CGAAA AUUGUA	CPSF4 AAUAAA
Dusp19			X
E2f2	X	X	X
Htra2			X
Ociad2		X	X
Perp	X		X
Prr9	X	X	X
Rtkn2	X		
Scarb1			X
Smarcd1	X	X	X

b

Prr9 : NM_175424.3 Mus musculus proline rich 9

AGTCCACATCCTTCATTGAATCTCATTGAAGATCAGGCTCTTCAAATCACCTGAAATGTCTTTCAATGAT
CAACAGTGCAAGCAACCATGTGTGCCACCCCCATGTCTTCAAAAGACCCAAGAAAAGTGCCAGGCACAAG
CTGAGGATGTGTGTGTCTCCTCATGTCAGGACCCCTGCCAAGATAAATGTCCACAACAAGCTCAGGAAGT
ATGTGTTTCTCAGTGCCAGGAGTTAAGCCAAGGAAACTGCCCACAACAAGGCCAAGATCCATGTCTACCT
CCAAGCCAAGACCAGTGCCTTCCTCAGTGTGCGGAGCCATGCCAGGAGCTAGCACAAACAAAATGTGTGG
AGGAGTTCCCACAGAAGGTACAGGAGAAGTGTTCATCCCAGAGCAAGGGGAAGTAGCTGCTCAAATGCCA
TCTGGAGTCAAGAAGATGGCCAACAGAAGAAAATACAACCCCAGTCCTACGGTGACCTTCTCCTGTGGGT
ATCTCTGTGTAAGATACTGTCTTGTCTCCTGACTTCCTCAGTATTCCAGGACTTGGTCTGTACCTCTGAA
GATAGTTCTCTGTATTTTATTAACCTCTGTGAATAAGTGTTCTCAGCAGCTGGCAGACGGTCAAGAGTAT
GGGAATGTTGTGGTTATTGAGGCAGACCATCAAAGCCTAACACAACTCTCCTGGGGCAAAACCTCACTTA
GCCCCTGGTACATAATGGCCAAGGATGCCTTTGTCCATCTTTCAGAGTGCAAGTATCTCTTTCAAGCCTC
AAGAGAATCTGAATTTAATGGCTTTATGGTGAGCATCACCACTACAATGAGTATGAAGAAATTGGTGATG
CTACAAAATCAAAAATGGTTTAATTTTGTTTGTGCATCCTAACCAAAAAAAAAAAAATAGCTGAACATTG
TAATGTGGTCTGTGCTTTGTAAAATTCAAATGAAATGTTTTAAAAGACATAATTCAAACAGCCTATAAGA
AACACAAGTTGGGATCTTTAGATCTTTGTCAGTCTTTGTGCTTTGTAGATGATCTGCTTTGGACCATTTG
GTGTGAGAGGGCTTTGGATACTATGATTCATCTTTAGTTTGTGTTTCTGGATAGGAGCTGGCTTCTCTC
CAACTTCCTTGCCTTTCCCCCTTGGTGGAAGTAAAATATAAATAAAGCTCTTTCTAAGGCTG

c

E2f2 : NM_001305399.1 Mus musculus E2F transcription factor 2

CATCAGTGACCTCTTCGACTCCTATGACCTTGGGGACCTGTTGATTAATTGAAGCGTCCTGCATCTGTCT
ACCTCCTCACAGACAGATTGACAGGCTCTCTGCCCAGATGGGGGACACCAGACACCAGGTGTCACCCTCC
GTCTGCGCGGCTCTCTTCCAGCCTTCCTTAGGGCGGAGGCCAGCTAGGGAGATGTGGAGGATTCGGCTGG
GGAGTGATATCAGGGCTAAGCCCTGACCCTCCTCTTCTGACCTCCGACCCCTTCATGAAGGACTACGGGT
GCAGGTGGCCATGTTCAGGGAAAGGGCTCACTGCCTCCTTCTGAGGGGCAGGGGGACCCTGACTTTTTCA
AGAAGCACTTAACGCCTTTGTATTTATTTCTAGTTCAGATGTGTTTGTTTGTCCACCCTGAGTTTTAGCA
GGGAGATTTGTTCTAGTTTCTCCTCAGACAGGCCCACCCACGCCCAGTGCCTAAGCGCACATCTGGGCTT
CCAGGGGTCTAGGCCAGCTCCTTGCCCCCCGTCCCCCCGTCCCCCCAGTGGTGCCTGGGTCTTGAGGGGA
CTATCCAGTTTGCTTGAATTAATTGCACTTAGGCTTTGTAATTTTAGAAAATGCCAGGCCCAGCCAAGGC
ACCGGCTTGGAATTCTCCATCTCCTCCATCCTAGAGAAAGTTCCAGTAGCTACTCTGGGGGAGGTAGCCA
AGGGGGCTGGCTTCCTATTGGATGTCTTATCTTTCCTGAGCCTCCCAATGTAAATGTAGGCCTTCAGGAA
TTAGCCCAGAAGAGCTCCTGGGAAAAGCAGGAGGCCACGCCCGTGCCTCCTAGATGCCCTTCCTCAGGAG
GTCAGAGACCTGGAAGGTGGAGGCAGAGAGGAGTGAGAAGAAAACAAGAAACACCCTGGGGTCTGCCCAT
GTTTCCCTGGGAGGATTATTTTTGTTTCTTGGTTGCAGTTGGAGGGGGAAGATTCCTTAAACACCCACTG
TCCCAAAGCCCTCACTTCACTTCCAAGGTCAGATGTGACAGGAGCTTGGTGACAGGTTTGCACCTTTCCC
CACTGGAGTGCTTCGGGGGTGAGGGGGCAGGGGTGGGGTGCTCTTCCCTGCAGACTAGGCCTCAGCAGCT
CATGACGGGATAGGCTGGCGCCTGCCACCTCTCCTGGCATGGGGATCTGGGAAGAGCTCTTGGTCTCTCT
TGTTCTGCCCATAGACTGACCATCCAGGGTTCCTGTGGTCAGGAGACGGCAAGAGTCCGCTTTTCTTCCT
CGCCTTTCCAGGCATGGAGAATGGGCTTAACCAAGGCCACGTGTCCAGAGAGGTGTACCCAGGGGTGACG
GGGTCTGAGAGGCCTTCCACCTACCCTCAGGGAGCCTGGGTTTCTCAGGGGCTCAGTGGGGCAGCACTTT
TTGCTTGTTGTTGTTTTAAGTTTGTAGCATTAAATTGATTTAAAAGATGAAGTTGGCTATGCCAGGTAGG
TCCGAGCTAGCTGACTGGGAATTTGAAGTTTGGATGTTGAGCTAATATGTACTGGTGCCCATTTGGGTTA
ATTTGTCTTATCCTCGAGTCAGCTTCCTGGTGCCACAAACTTAGTCCTGTCCGAGGAGTGGTCAGCGACT
CACCCCCCCCCACACACACTTGAAATTTGTCTATTTTTACACCGAGTGTCCAACCTTTTGACATATGTGA
CCTCGTCGTCTCTAAAGTTCACCCTCAGGAACTGCATAGATAAGCTACCAAAAAAAAAAAAAATCTCATG
ATGTTTGAAGTACGTTTACAACTCTGTGTTGGGCTGTGTTCGTAGGTGTCTGCGACCCGTGCTGCAGGTT
AGATACACCTGCTGCAGAGATGTCCTTGGTCTCTACCAAGCGAGGGGGGTTTCTCAGACCACCCAGAAAG
CCCAGTGTGCTCTAGACACAGGGGGTCCCTTGAGTGATGAGGGTGCTACCTGAGATCTGGTTTGATGCAC
ATGAGGCCGGAGACAGCATTGCTGTTTCCTGGTCTTTGCCGTTTAGCTGTGCGCCAACAGAGATATCTGA
CCTCTGTGCCTTGGGCTCTGGGTGGAGGAGAGCTTGGCTTAGCGAGAAGAGGGCTGCCATTCCTGTCTGG
TGTGAGGTTAAGAATACAGTCCAGATTCAAGTTCAGTCAAAAGACCCAGCGCAGGGATGGATGGAGGCTT
CCCCAGTCTTTTTGATCTCTGAGCTCTTTCTTATCAACCCTGCCTGGTCCAGGGGCTGGACCTCCCGGGA
GAAGCCCGAGGCTAGCGTGGGAACAAGCTCACACCTCTTTCTTGCCAATGATACTTGAGACCTCTTTAAG
TTCCTTAGAGTGCTTTAAAAGAACGGGGTGTACCCCTTTTGGAACACTGTTTTGAGTGGTATGTCTTTTA
GGCCCTAGGGAAACGTTCATGCTAGGGTGGGAAAGCTACCAGTTTCTTTCTGAGGGGAATACTTTCTTCA
GGGAAGTTTCCCAGCCCTACCAGTGTCCCCTGCTGATATCCCTGGGGACTCCAGAGCATTTAGCAGAATT
AAACTTGTAAATAGGCTTGGAGATGCACCATTGAAGATGTCAGGCTGAGTCCCTTCTCTGTGTGGCTGTG
AGGAGTGGGAGTTTGGGGGAGTTTGTAAACTATTTCAGGGGGTGCAGTGTGGTTTCCCCTCCCTGTGAGG
CCTCAGCCTCTCTTCTGCACAGATTCCGTGTTGTAAAGAGTTTGCTCATGCCGCCATCGGTGTGCTATAG
CTTTAACGCTTCTGAAACCTCTGGGGTTGATTAGAAGCCGGGGCTTAAGTTTCCGTCGTACCCAGAGGCC
CCACCTCACCCAGGTCGTCTCTTCTCCCACAAGGCTCTTCAGGGACCGTGGTGGCTACAGGTCTGGCCAG
CCTCTGGCTCCTAGGAAATATGCAGTCTTGTGGGGTTTGATCATTTTGAAGATACCTGGGGGTGGGAAGG
TCTGGAGTCCCTGGGTTGGGACTCCATGTGGGTCACCTACCTCCTGGTTTCACCCAAAGTCCTGCCTTGA
ACCACCAGCAAGTTTCATTCTGTTGTGATAATTGGGCATTAAATAATGGCGGCTCTGGTAAAAAAAAAA

d

Smarcd1: NM_031842.2 Mus musculus SWI/SNF related, matrix associated, actin dependent regulator of chromatin, subfamily d, member 1

CTTCAGTCCCAGTGCAGGGACCTCAAGACGATGACTGATGTGGTGGGTAACCCGGAAGAGGAGCGTCGTG
CTGAGTTCTACTTCCAGCCCTGGGCTCAGGAGGCTGTGTGCCGATACTTCTACTCCAAGGTGCAGCAGAG
GCGGCAAGAGTTAGAGCAAGCCCTGGGAATCCGAAACACATAGGGCCTCTGTGGCCCTAGCCTGGCTGCA
CCGATTCCTTGGGCCCTGTGCTGCCTGCCTCAGTGTACCTGTCTTGGTCTTGCTTGAGGCATTCCAGGGG
ACTTGGCTTCAGGACAGTGTCACAATGAAGAGGGTGTCACATTTCTGTCTCACAGTCACCTGTTATCCCG
TCCTGTACCCCAGTCGTCCCCCGTCCCGTCGTGTCCCCCCCTCACCCCACCCCGCCTCAGCTCCTCCCCA
TCAGGCTCCTGTGTGCCTCTACCTCCCTATCCTACATAGGACCTCTAGATAGTGTTAGAGAACCACAGAG
TGGGGGCCTCCTGAGGTCAGGTGGTCTTGAGGGAGACCAGCTACACTGATCCTGCCCTTGTCAGGAGACC
TAGGCCTTGGGAGCTATCCCTGTCTGAGCCTCAGGCCTAGGGCAGTCTGTAAGCTAGCTGACCTTGGCCC
TCCCGGTAGCTTGACTTCTTCCCTCCCCTCCGCAGGTTGGGGCAGAGGCTCCTTTACCTCTGGCAGTAAA
GGAGCCTGGGCTTCACTGAGCCCCGGGTTGGTCCCCTGCCCTCTGGACTTAACCTGCTGTCTCAGTGTCC
TCTGACCCCTTAGGGGTCCATGTCAGTATTGGAGTGTGTGTTGAATTGTTGCTCCCTCCCACACACTCCC
GTAGCCGCCCAGTTTAGGATTTCCCTACACCTGCCCTAACCCACGCTTTTGGGTTGGGGATCTTGCCTTT
CCTTGTCATTCCCAGCAGAGACTGTTCCTTCCTGCTGTTAGAGGAGTGGCTTGTTTATTCACTCCACCCT
GCCCCCTCCTGTAAATGGAGAAACAGGCCTGAAATCAAACGGGTAAAGCCCTAGGCCATCCCTGTCTTCC
TGTCCCATGTCTGCCCAGTTGAATCCCACTGGTGGCTTCCCGGGCACTGAGGAGTAAAAGCGCCTAGGGC
TGGAGAATAGGTCTGAAATGGGTTTGTGACTCCCCACCCCCTGCCCTGCCCTCAAAGCTTCAGACCCCTC
AGGGAGCAGCAGGATGTGGGATCGAGGCCCCTTGGGACAGATGCTTTGAATCTTCCAGGGAAGCCTCCGA
TTCTTCCAGGTTTGTCACCCGGAGTTAGCATGTCCCAGGCTCGCAGACAACACTGCAGGGTGGGAGACAG
CTGGGCACAGGGGGATTCTGTTGAGCATGGGCTCTGAACCCACAGAACTGACAAAGCCCCTGCTTCCCCA
CCCCCACCTCAGGCTCCTGCGAGCAGTGCTCCTGCACCCTTCCCAGCCTGTTCTGTACTGGGGACAGCAG
TCTTCTCCCTGTCCTCCCATGTCCTATATCCACCCCTCCCCTTGGAAGGTCCTCCCCACAGTGACACTGG
ACAGCCCTGGGGCAGCTGAGCCCCAGCCTGGCTTCTGGCTGGAAGCGCGATGAGGAGACTTAGCACTCCA
CAGTGTCCCTGGTGGTAACTGTTCTTATTAACTGATTGTGTTTTGTTTTGTTTTGTTTTGTTTTCATGGA
CCAAAATTTTTTTTGTACTGTCTCCTTAACTGATGTCACCCAGTTTTAATAAAAGACTTCTAAAGAGCAG
GTC

e

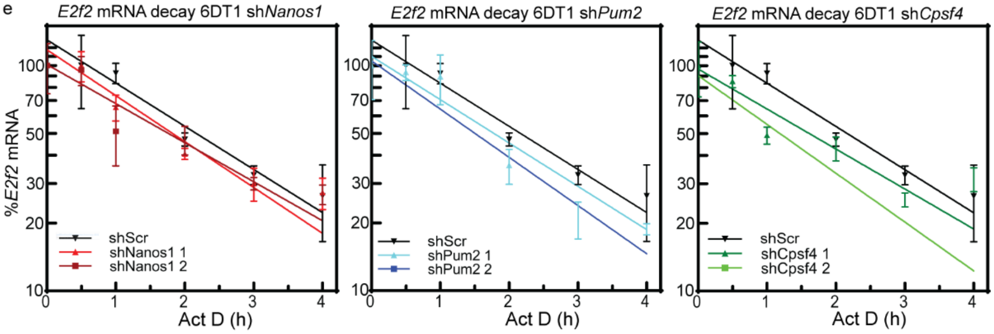

f

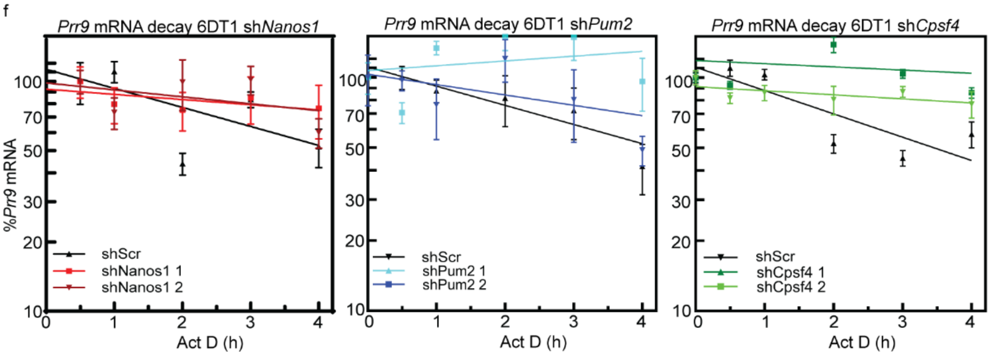

Figure S3. Sequence and half-life analysis of differentially expressed transcripts in RNA-binding protein knockdown lines.

a, table showing the presence of a PRE, NRE, or CPSF4 binding elements within the 3'UTR of each gene listed where x = presence of element. b-d, 3'UTR sequences with PRE, NRE, CPSF4 binding elements highlighted (red: stop codon, purple: PRE, blue: NRE, yellow: CPSF4 binding element) in the *Prr9* 3'UTR (b), *E2f2* 3'UTR (c), and *Smarcd1* 3'UTR (d). e, graphs showing actinomycin D (actD) time course to measure *E2f2* RNA half-life in 6DT1 shN*anos1* (red), sh*Pum2* (blue), sh*Cpsf4* (green) vs shScr. f, graphs showing actD time course to measure *Prr9* RNA half-life in 6DT1 sh*Nanos1* (red), sh*Pum2* (blue), sh*Cpsf4* (green) vs shScr.

Supplemental Figure 4

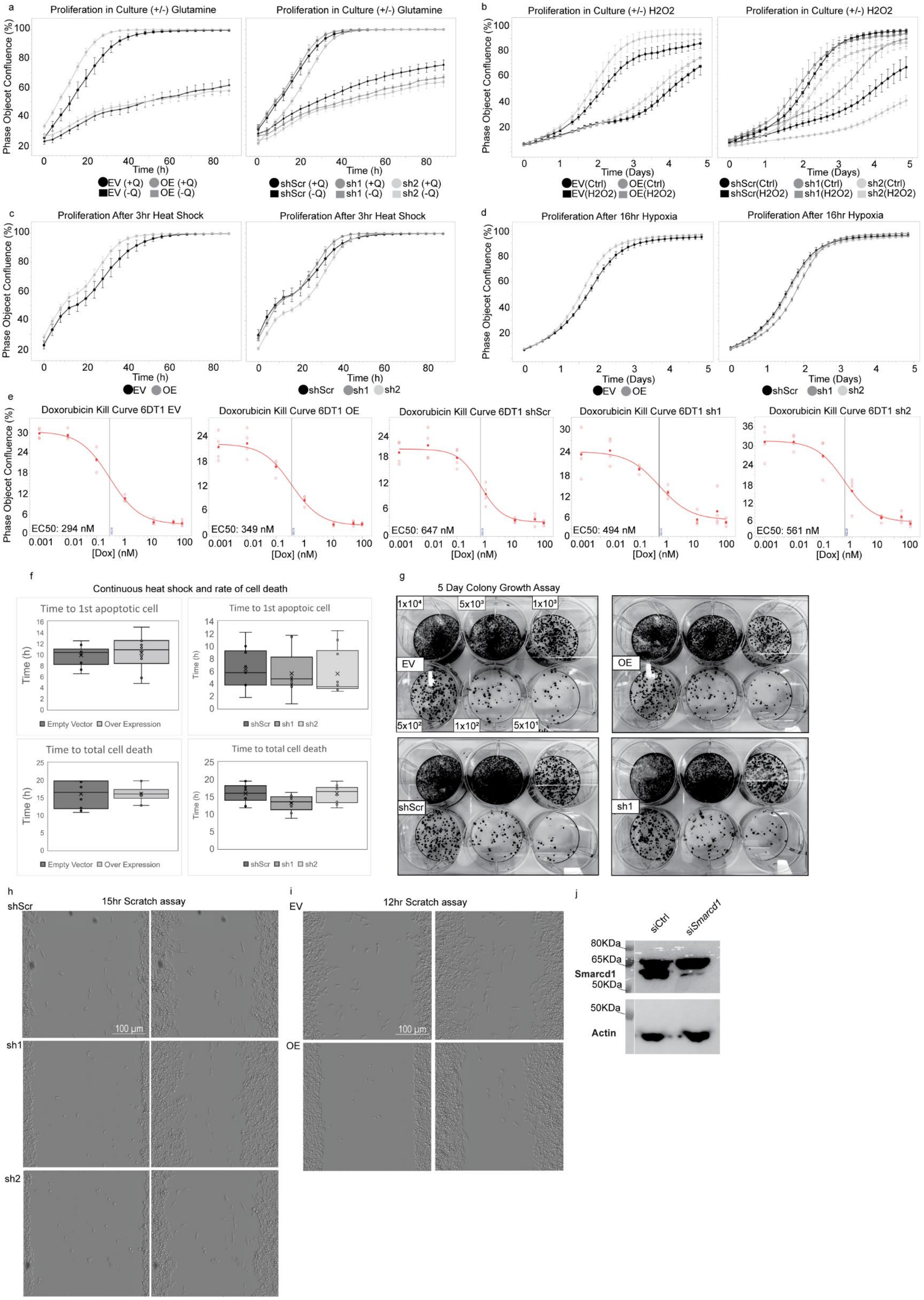

Figure S4. Altered *Smarcd1* expression does not impact cell response to stress.

a-d, graphs showing phase object cell confluence over time following treatment with "no glutamine" media (a), treatment with H_2O_2 (b), 3 hour heat shock at 42°C (c), and 16 hours in hypoxic 1% oxygen conditions (d) (n=4). e, kill curves showing treatment curves and EC50 values for each cell line after 24 hour treatment with a serial dilution series of doxorubicin (n=4). f, Box plots showing time to appearance of the first apoptotic cell and time until all cells were apoptotic during 20 hours of continuous heat shock (n=10), mean indicated by "X" and median by inner quartile line . g, photographs of colony growth assays with cells seeded in 6-well plates at limiting dilutions. h, representative images of migration assays at time point 0 and at 15 hours in 6DT1 *Smarcd1* KD lines vs shScr control. i, representative images of migration assays at time point 0 and 12 hours in 6DT1 *Smarcd1* OE lines vs EV control. j, western blot showing Smarcd1 and Actin protein levels after transfections with control "siCtrl" or *Smarcd1*-targeted "siSmarcd1" siRNA.

Supplemental Figure 5

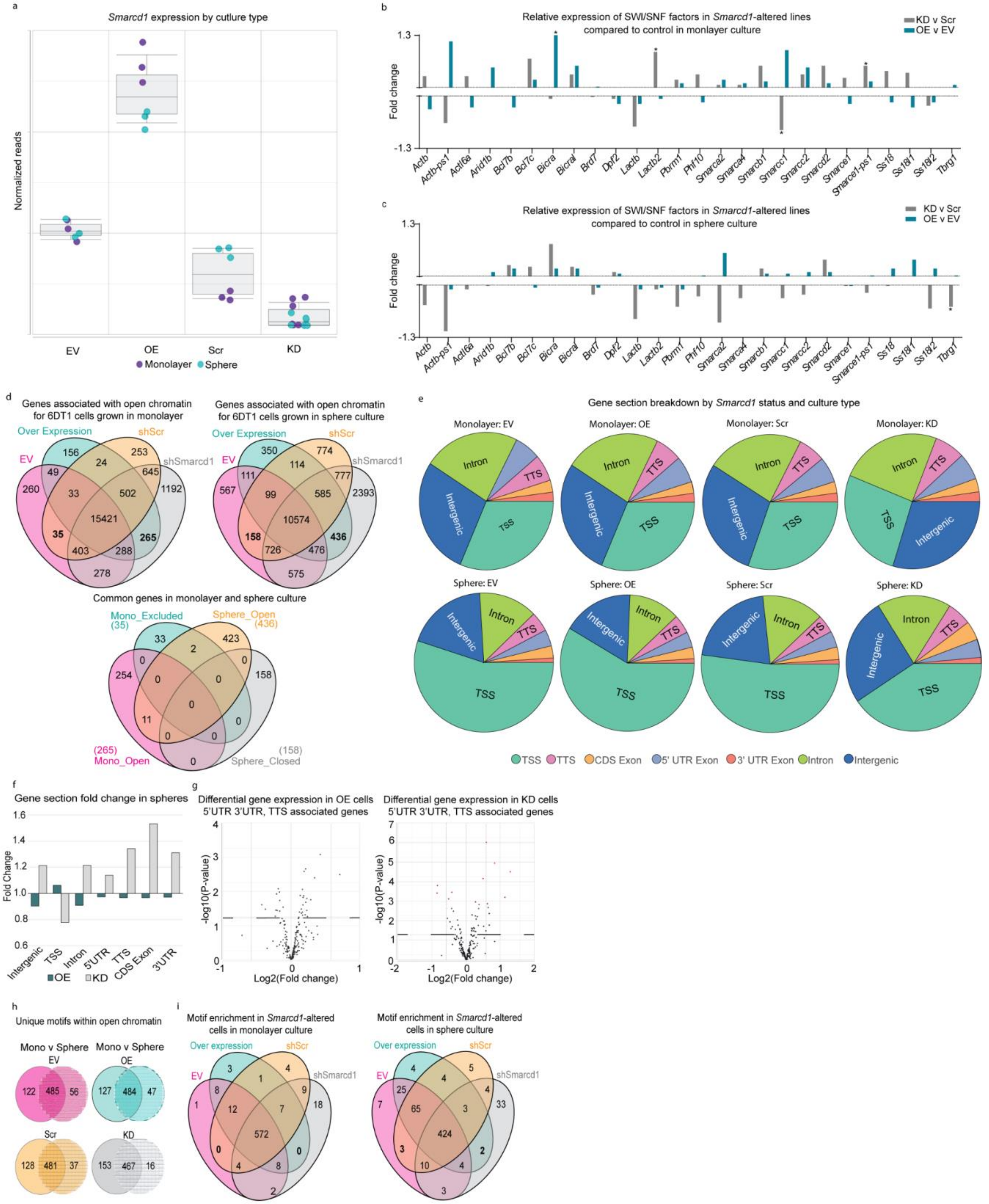

Figure S5. Impact of altered *Smarcd1* expression on gene expression and chromatin accessibility.

a, RNA-seq data showing *Smarcd1* mRNA levels in 6DT1 EV, OE, Scr, and KD lines in monolayer and sphere culture. b-c, RNA-seq data showing relative abundance SWI/SNF factor mRNA in *Smarcd1*-altered cells vs controls in monolayer (b) and sphere culture (c). d, Venn diagram analysis of genes associated with regions of open chromatin for each cell line according to culture type. e, pie charts showing the relative percentage of each gene section found in sequenced open chromatin. f, graph showing fold change in the enrichment of specific gene sections (Intergenic, Transcription start site or TSS, Intron, 5 prime untranslated region or 5'UTR, Transcription termination sequence or TTS, coding sequence or CDS, and 3'UTR) in open chromatin for *Smarcd1*-altered cells vs control lines grown as spheres. g, volcano plots showing changes in gene expression for genes with 3'UTR, 5'UTR, and TTS enrichment in open chromatin in monolayer culture (red=fold change >1.5, blue=fold change <1.5). h, Venn diagram analysis of unique motifs enriched in open chromatin for each cell line in monolayer vs sphere culture. i, Venn diagram analysis of motifs enriched in open chromatin for each cell line in monolayer or sphere culture.

Supplemental Figure 6

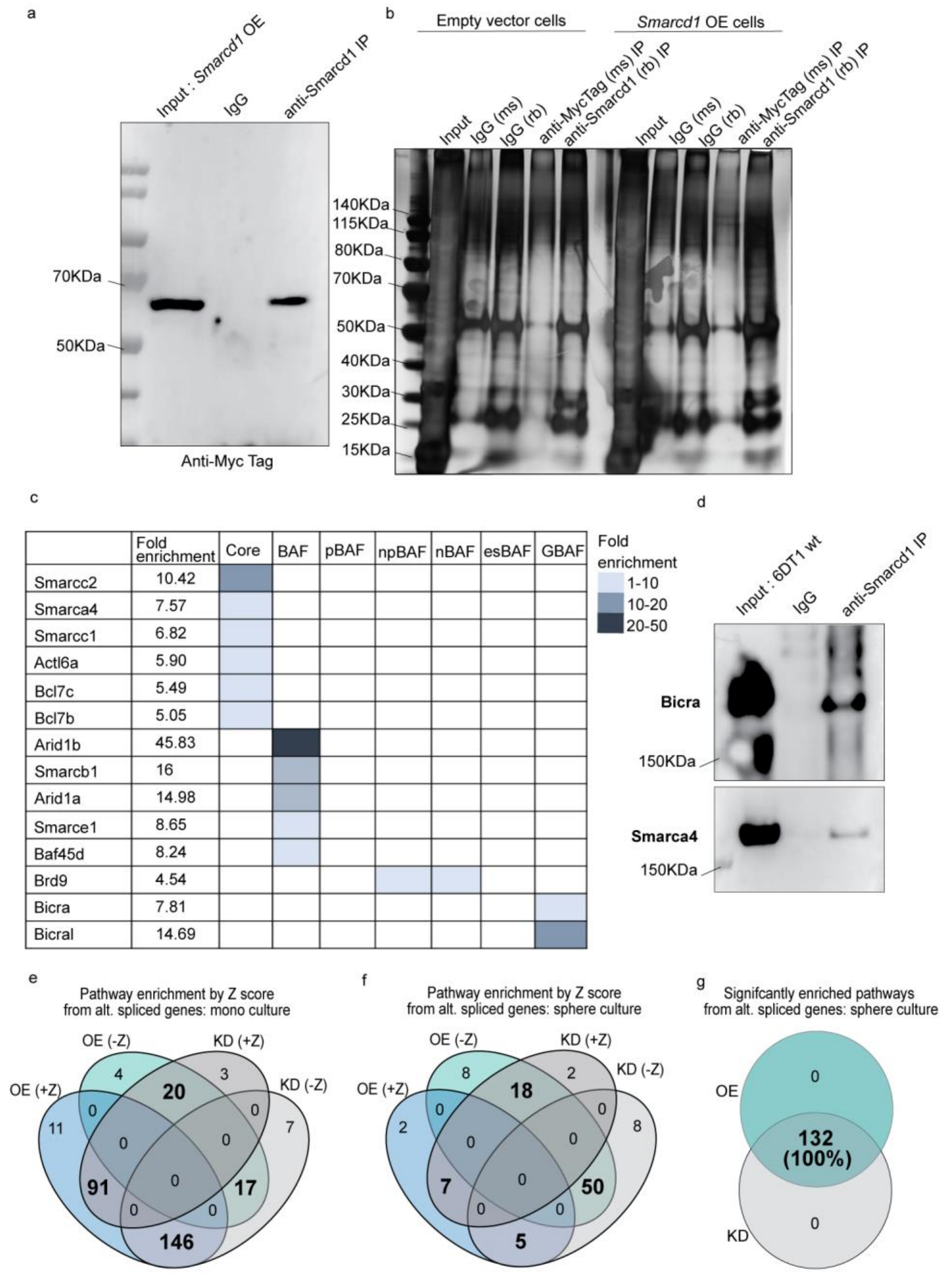

Figure S6. Smarcd1 interacts with BAF complex members and alters splicing programs in sphere culture.

a, western blot showing efficiency and specificity of Smarcd1 immunoprecipitation (IP) from 6DT1 *Smarcd1* overexpression (OE) cells. b, silver-stained gel showing relative protein abundance in input, IgG control, Myc-tag, and Smarcd1 antibody IP samples. c, table showing fold enrichment of BAF complex members in *Smarcd1* OE vs EV. d, western blot showing that Bicra and Smarcd4 immunoprecipitate with Smarcd1. e-f, Venn diagram analysis of pathway analysis Z scores in *Smarcd1* OE and KD lines vs controls in monolayter “mono” culture (e) and sphere culture (f). g, Venn diagram analysis of pathways significantly enriched ($p<0.05$) by alternatively spliced genes in OE and KD lines from sphere culture.

Supplemental Figure 7

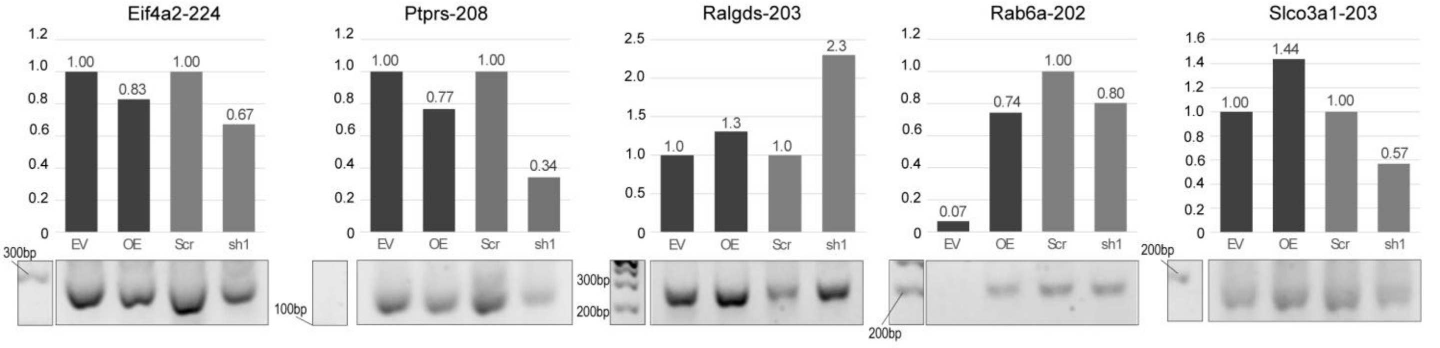

Figure S7. *Smarcd1* expression levels alter the abundance of specific transcripts.

Densitometry of gel images showing PCR amplification of specific transcripts in RNA isolated from 6DT1 Empty Vector (EV), *Smarcd1* Over Expression (OE), shScr (Scr), and Knockdown (sh1) cells.

Supplemental Figure 8

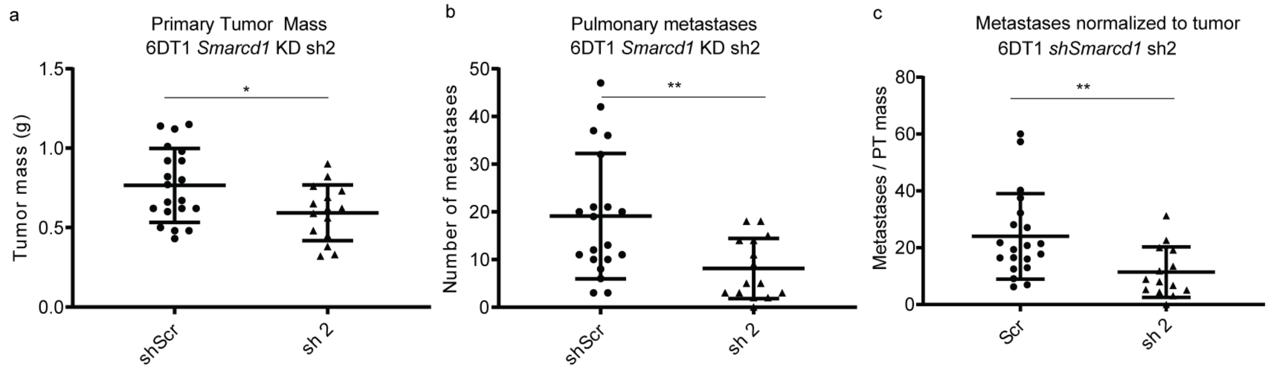

Figure S8. *Smarcd1* KD decreases metastasis in an orthotopic mouse model.

a-c, analysis of primary tumor (PT) weight (a), number of lung nodules (b), and number of lung nodules per gram of PT per mouse (c) 28 days after orthotopic injection of 6DT1 shScr control and shSmarcd1 line 2 (2 combined experiments, 10 mice per experiment).* = p<0.05, ** = p<0.01.

Supplemental Figure 9

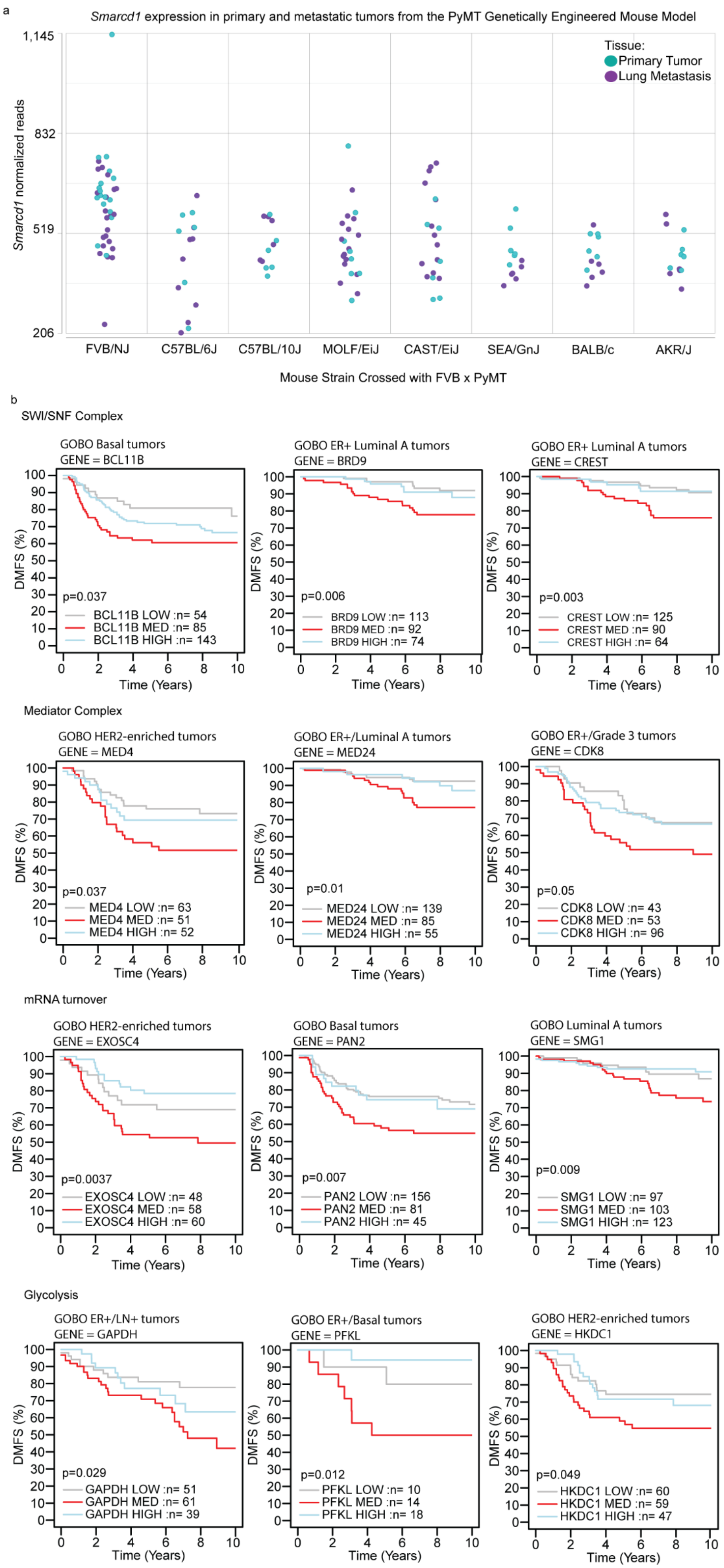

Figure S9. Essential expression-restricted metastasis modifier genes exist throughout cellular processes and clinical subtypes.

a, graph of *Smarcd1* RNA levels from RNA-seq data of matched primary tumor (PT) and lung metastasis pairs from the FVB x PyMT GEMM crossed to several mouse strains. Each point is data from 1 mouse, green=PT tissue, purple=metastatic tissue. b, representative Kaplan-Meier analysis plots showing stratification of distant metastasis-free survival (DMFS) for patients with different subtypes of breast cancer by expression level of the indicated genes (grey=low expression, red=medium expression, blue=high expression).

Figure S10: Original Blots /Gels. The original uncropped gel or blot image is shown for each figure in the main text.

Figure 5a

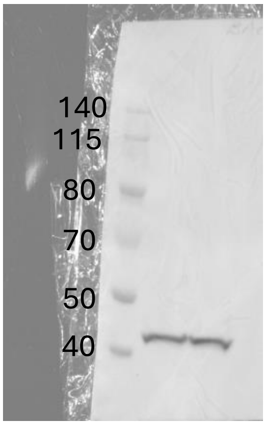

Beta Actin

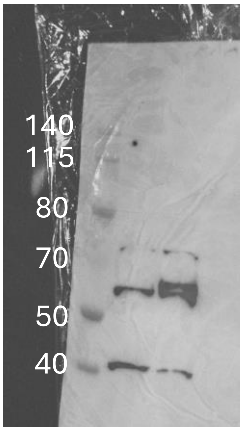

Smarcd1

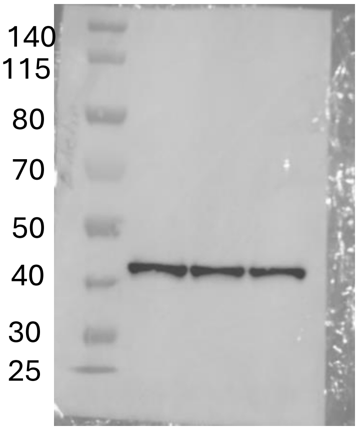

Beta Actin

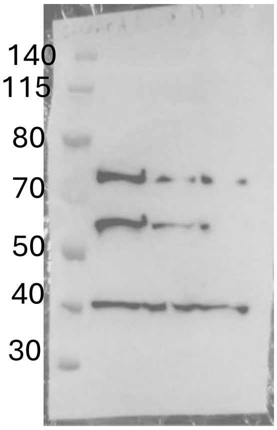

Smarcd1

Figure S1

6DT1 Pum2

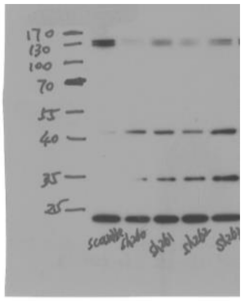

4T1 Pum2

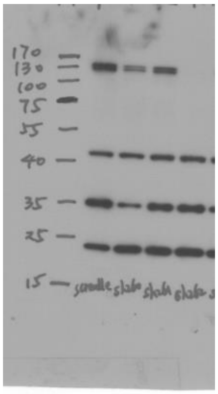

6DT1 Cpsf4

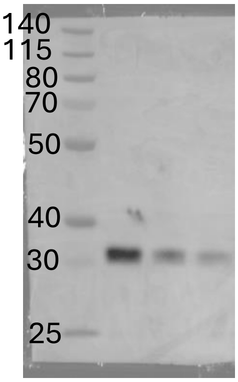

6DT1 Cpsf4

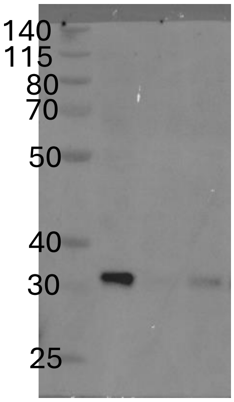

6DT1 Actin

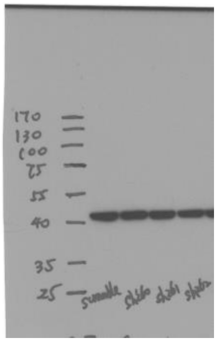

4T1 Actin

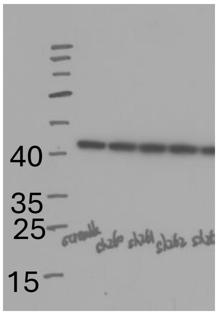

6DT1 Actin

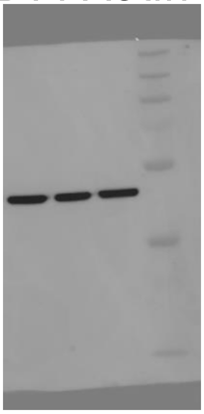

6DT1 Actin

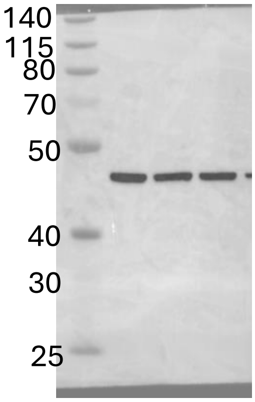

Figure S4j

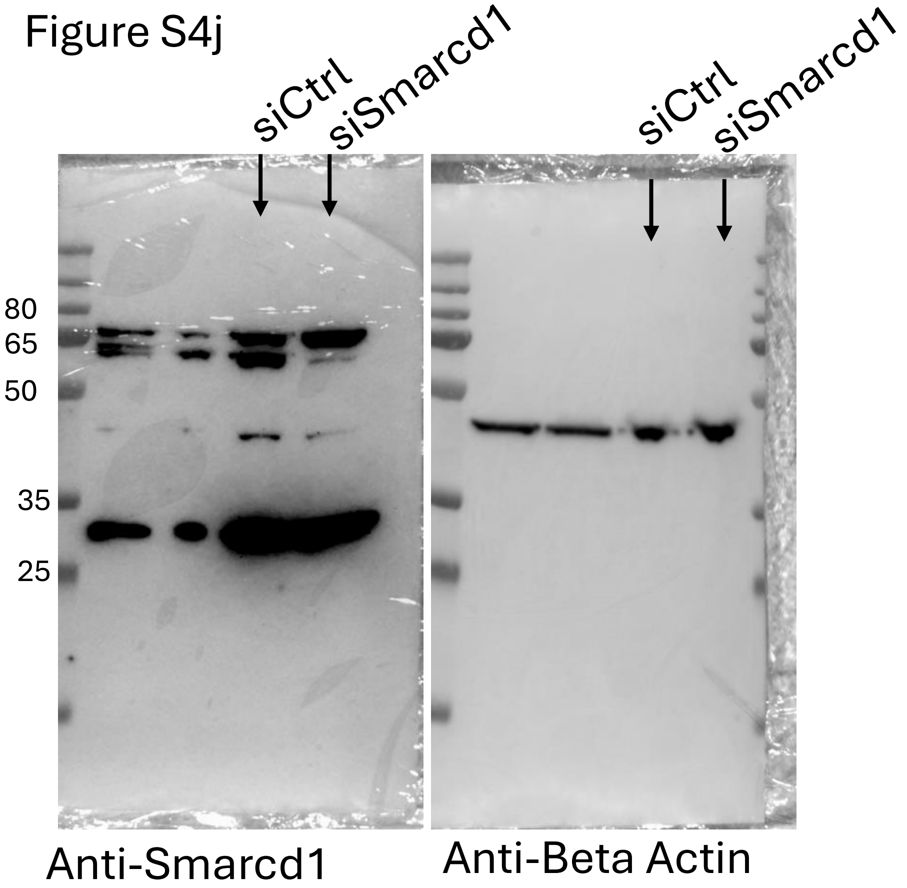

Figure S6d

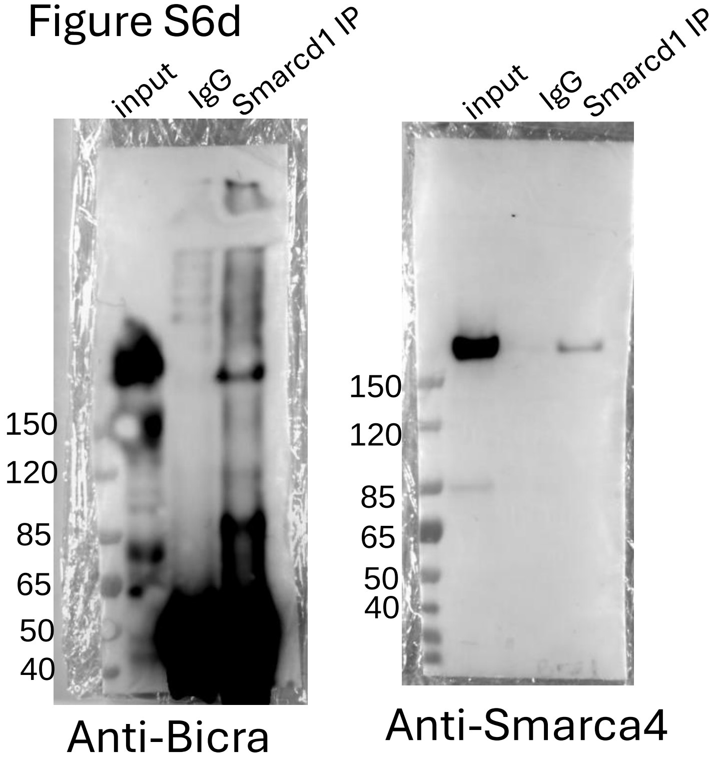

Figure S7

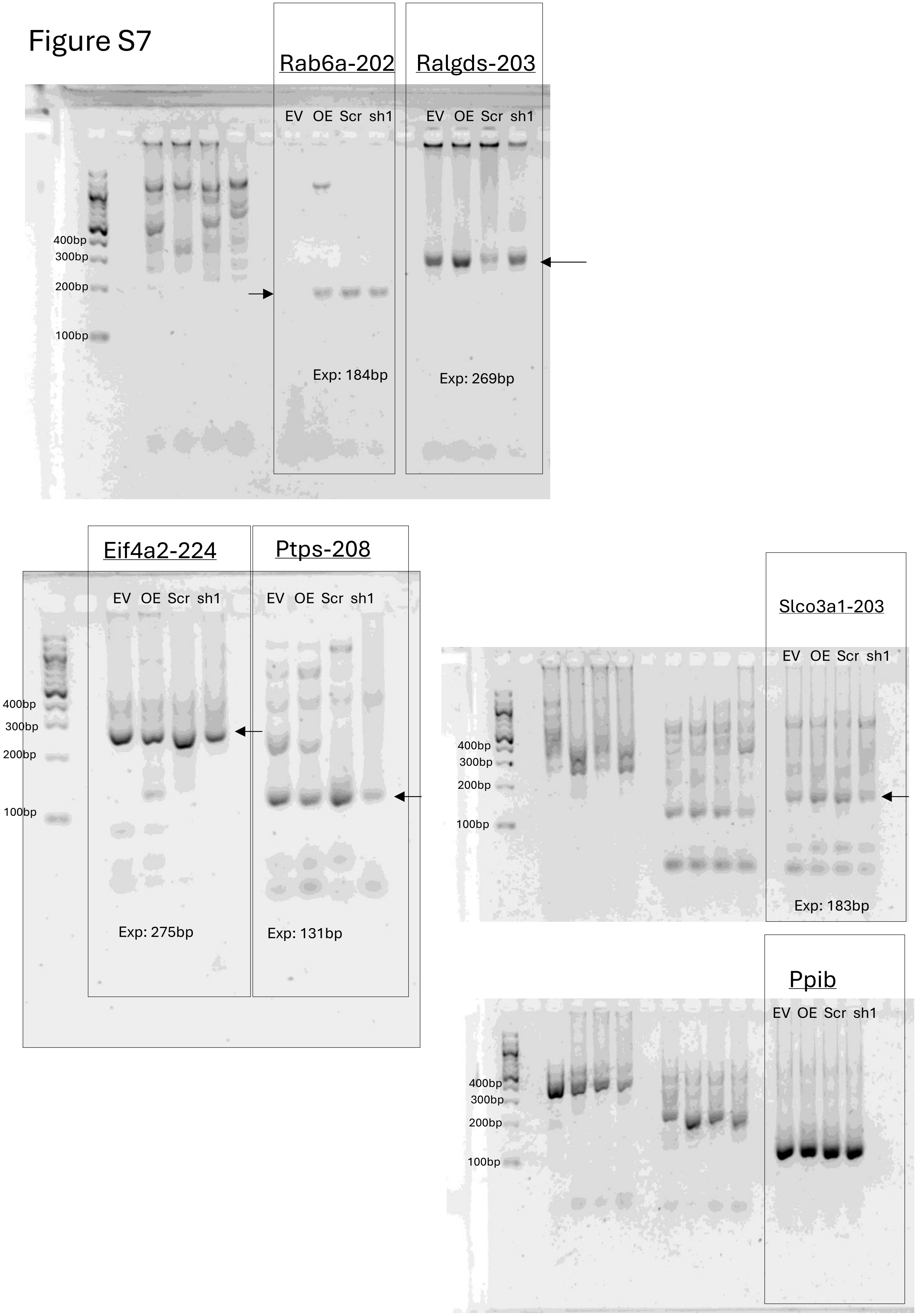
Rab6a-202
EV OE Scr sh1
Exp: 184bp
Ralgds-203
EV OE Scr sh1
Exp: 269bp
400bp
300bp
200bp
100bp
Eif4a2-224
EV OE Scr sh1
Exp: 275bp
Ptps-208
EV OE Scr sh1
Exp: 131bp
Slco3a1-203
EV OE Scr sh1
Exp: 183bp
Ppib
EV OE Scr sh1

Supplemental Tables

Table S1: E2f2, Prr9, and Smarcd1 mRNA half-life summary table.

Trasncript half-life					
**Transcript**		**Knock down cell line**			
		**Control**	**NANOS1**	**PUM2**	**CPSF4**
E2f2	sh1	1.6 h	1.7 h (n=1)	1.5 h (n=1)	1.7 h (n=1)
	sh2		1.5 h (n=1)	1.4 h (n=1)	1.4 h (n=1)
Prr9	sh1	3.4 h (n=6)	>6 h (n=2)	> 5.5 h (n=2)	>6 h (n=2)
	sh2		>6 h (n=2)	>6 h (n=2)	>6 h (n=2)
SmarcD1	sh1	2.9 h (n=11)	>6 h (n=4)	>6 h (n=5)	>6 h (n=4)
	sh2		>6 h (n=3)	>6 h (n=4)	>4.8 h (n=4)

Table S2: Smarcd1-interacting proteins.

Accession	Gene	OE Smarcd1/ EV Smarcd1	Annotation
E9Q4N7	Arid1b	45.83	BAF
Q9Z0H3	Smarcb1	16.00	BAF
E9QAQ7	Arid1a	14.98	BAF
Q8CHH5	Bicral	14.69	BAF
Q61466	Smarcd1	11.81	BAF
Q6PDG5	Smarcc2	10.42	BAF
O54941	Smarce1	8.65	BAF
Q05D44	Eif5b	8.30	BAF
D3Z5N6	Dpf2	8.24	BAF
F8VPZ9	Bicra	7.81	BAF
Q3TKT4	Smarca4	7.57	BAF
P97496	Smarcc1	6.82	BAF
Q9Z2N8	Actl6a	5.90	BAF
Q921K9	Bcl7b	5.49	BAF
A0A0U1RNX8	Bcl7c	5.05	BAF
A0A0R4J175	Brd9	4.54	BAF
F8WI88	Pcnp	14997.00	CELL CYCLE
Q6A065	Cep170	4734.47	CELL CYCLE
Q65Z40	Wapl	4579.48	CELL CYCLE
Q60865	Caprin1	1.19	CELL CYCLE
O55047	Tlk2	13476.92	CHROMATIN REMODELING
Q8K2H1	Pphln1	9928.54	CHROMATIN REMODELING
Q80W03; Q8BU11	Tox3; Tox4	8780.74	CHROMATIN REMODELING
P10711	Tcea1	6163.53	CHROMATIN REMODELING
Q80Y32	Hmgxb4	1623.78	CHROMATIN REMODELING
Q9D8Y8	Ing5	33.72	CHROMATIN REMODELING
Q60953	Pml	27.16	CHROMATIN REMODELING
G3UWZ0	Baz1a	9.45	CHROMATIN REMODELING
Q7TNV0	Dek	8.69	CHROMATIN REMODELING
A0A1L1STE4	Ilf3	8.07	CHROMATIN REMODELING
P17095	Hmga1	7.66	CHROMATIN REMODELING
Q9R190	Mta2	6.97	CHROMATIN REMODELING
Q60972	Rbbp4	6.76	CHROMATIN REMODELING
Q91WC0	Setd3	6.45	CHROMATIN REMODELING
Q9CXY6	Ilf2	5.92	CHROMATIN REMODELING
O09106	Hdac1	5.79	CHROMATIN REMODELING
Q9CQJ4	Rnf2	5.44	CHROMATIN REMODELING
Q9WTU0	Phf2	5.27	CHROMATIN REMODELING
Q3TUF7	Yeats2	5.23	CHROMATIN REMODELING
A0A384DV79	Hmga1	5.22	CHROMATIN REMODELING
Q8BSY0	Asph	5.06	CHROMATIN REMODELING
Q8CHV6	Tada2a	5.01	CHROMATIN REMODELING

Accession	Gene	OE Smarcd1/ EV Smarcd1	Annotation
P63158	Hmgb1	79298.08	DNA DAMAGE
P11103	Parp1	40942.05	DNA DAMAGE
Q3UMU9	Hdgfl2	3072.18	DNA DAMAGE
Q2VPQ9	Meaf6	11.12	DNA DAMAGE
A0A087WQB0	Morf4l1	11.10	DNA DAMAGE
P97868	Rbbp6	10.23	DNA DAMAGE
P30681	Hmgb2	9.43	DNA DAMAGE
Q7TPD0	Ints3	8.88	DNA DAMAGE
Q3TCH7	Cul4a	8.14	DNA DAMAGE
Q61216	Mre11	7.27	DNA DAMAGE
Q8BZH4	Pogz	6.16	DNA DAMAGE
Q62318	Trim28	6.13	DNA DAMAGE
Q8CHI8	Ep400	5.98	DNA DAMAGE
Q9R0Q4	Morf4l2	5.80	DNA DAMAGE
Q8C0V0	Tlk1	5.79	DNA DAMAGE
B1ASU9	Tlk2	5.75	DNA DAMAGE
Q9JI44	Dmap1	5.42	DNA DAMAGE
A0A0R4J1I3	Men1	0.62	DNA DAMAGE
P39054	Dnm2	18877.88	EXOCYTOSIS
F8WIA1	Clip1	5118.81	EXOCYTOSIS
P17427	Ap2a2	884.75	EXOCYTOSIS
Q6KAR6	Exoc3	10.82	EXOCYTOSIS
Q3TPX4	Exoc5	10.00	EXOCYTOSIS
Q5ND34	Wdr81	6.97	EXOCYTOSIS
Q9D4H1	Exoc2	6.18	EXOCYTOSIS
P62821; Q9D1G1; P5	Rab1a; Rab1b	6.03	EXOCYTOSIS
Q8BJF9	Chmp2b	5.96	EXOCYTOSIS
Q6P1Y9	Exoc1	5.76	EXOCYTOSIS
O35250	Exoc7	5.38	EXOCYTOSIS
P17809	Slc2a1	10091.67	METABOLISM
P35492	Hal	9223.96	METABOLISM
P06151	Ldha	7202.05	METABOLISM
P55200	Kmt2a	2093.32	METABOLISM
Q61176	Arg1	19.74	METABOLISM
P12382	Pfkl	14.89	METABOLISM
A0A0R4J0B4	Cmas	10.68	METABOLISM
P40142	Tkt	9.74	METABOLISM
Q9CZW4	Acsl3	9.69	METABOLISM
P24270	Cat	9.66	METABOLISM
Q9Z148	Ehmt2	8.26	METABOLISM
P52480	Pkm	7.37	METABOLISM
Q99LB2	Dhrs4	7.27	METABOLISM
Q9DCN2	Cyb5r3	7.11	METABOLISM
P05064	Aldoa	6.85	METABOLISM
P35486	Pdha1	6.68	METABOLISM
Q91VA7	Idh3b	6.60	METABOLISM
Q9WUM5	Suclg1	6.57	METABOLISM
Q9DBG6	Rpn2	6.43	METABOLISM
P56379	Atp5mpl	6.34	METABOLISM
Q9CZN7	Shmt2	6.27	METABOLISM
P54071	Idh2	5.89	METABOLISM
Q8QZT1	Acat1	5.61	METABOLISM
P62897	Cycs	5.31	METABOLISM
P47740	Aldh3a2	0.00	METABOLISM
Q64437	Adh7	0.00	METABOLISM

Accession	Gene	OE Smarcd1/ EV Smarcd1	Annotation
Q921T2	Tor1aip1	6433.35	NUCLEAR LAMINA
Q80XP8	Fam76b	6421.25	NUCLEAR SPECKLE
P61514	Rpl37a	7.14	RIBOSOME
O35130	Emg1	6.72	RIBOSOME
P62843	Rps15	5.73	RIBOSOME
P67984	Rpl22	5.57	RIBOSOME
Q9JJI8	Rpl38	5.32	RIBOSOME
Q7TQK4	Exosc3	5089.38	RNA EXOSOME
Q921I9	Exosc4	6.99	RNA EXOSOME
Q9CSH3	Dis3	6.56	RNA EXOSOME
Q9DAA6	Exosc1	6.11	RNA EXOSOME
Q9D753	Exosc8	6.04	RNA EXOSOME
Q9CRA8	Exosc5	5.75	RNA EXOSOME
Q9D0M0	Exosc7	5.64	RNA EXOSOME
P97350	Pkp1	26156.04	RNA REGULATION
Q8VDM6	Hnrnpul1	44.02	RNA REGULATION
Q80Y44	Ddx10	10.36	RNA REGULATION
Q9QY15	Ddx25	5.83	RNA REGULATION
P62488	Polr2g	35.16	RNA SYNTHESIS
Q8CB77	Eloa	18.09	RNA SYNTHESIS
O35134	Polr1a	15.00	RNA SYNTHESIS
P62869	Elob	8.48	RNA SYNTHESIS
Q5SUS9	Ewsr1	5.51	RNA SYNTHESIS
Q8BFX0	Polr2k	5.29	RNA SYNTHESIS
Q6NXI6	Rprd2	0.00	RNA SYNTHESIS
Q61171	Prdx2	28749.39	ROS RESPONSE
Q9CQQ8	Lsm7	12703.37	SPLICING
Q9DAW6	Prpf4	7597.72	SPLICING
Q8C1D8	Iws1	4847.29	SPLICING
Q3USH5	Sfswap	4089.57	SPLICING
P98203	Arvcf	21.89	SPLICING
E9Q5K9	Ythdc1	20.69	SPLICING
Q8BQ46	Taf15	12.88	SPLICING
Q8BIQ5	Cstf2	12.41	SPLICING
P60335	Pcbp1	8.94	SPLICING
Q9QZH3	Ppie	8.80	SPLICING
Q99LF4	Rtcb	8.47	SPLICING
Q8JZX4	Rbm17	8.09	SPLICING
Q9CQI7	Snrpb2	7.93	SPLICING
P62315	Snrpd1	7.54	SPLICING
G5E8I8	Cherp	7.28	SPLICING
Q99MR6	Srrt	7.20	SPLICING
P62960	Ybx1	7.12	SPLICING
Q8K4P0	Wdr33	6.96	SPLICING
Q5U4D9	Thoc6	6.68	SPLICING
P62311	Lsm3	6.55	SPLICING
Q921F2	Tardbp	6.46	SPLICING

Accession	Gene	OE Smarcd1/ EV Smarcd1	Annotation
Q922U1	Prpf3	6.40	SPLICING
Q99LI7	Cstf3	6.35	SPLICING
Q9QXK7	Cpsf3	6.26	SPLICING
Q99LC2	Cstf1	6.23	SPLICING
F6RJ39	Acin1	6.17	SPLICING
Q9D0W5	Ppil1	5.91	SPLICING
Q8C2Q7	Hnrnph1	5.86	SPLICING
P62313	Lsm6	5.83	SPLICING
A0A0R4J0J6	Thoc5	5.77	SPLICING
A0A3B2WCR6	Snrpc	5.56	SPLICING
Q9CQF3	Nudt21	5.50	SPLICING
Q8QZY9	Sf3b4	5.39	SPLICING
Q9CWK3	Cd2bp2	5.30	SPLICING
A0A0N4SUZ3	Lsm8	5.28	SPLICING
Q99J95	Cdk9	5.19	SPLICING
O35841	Api5	5.13	SPLICING
Q99M28	Rnps1	5.12	SPLICING
P63154	Crnkl1	5.11	SPLICING
Q91VM5	Rbmxl1	5.09	SPLICING
P62307	Snrpf	5.06	SPLICING
Q8K194	Snrnp27	5.02	SPLICING
Q921M3	Sf3b3	5.02	SPLICING
A6H619	Phrf1	2.57	SPLICING
Q8C5J0	Sp2	2919.85	TRANSCRIPTION
Q6KAQ7	Zzz3	9.73	TRANSCRIPTION
Q9JMG1	Edf1	6.18	TRANSCRIPTION
P57776	Eef1d	7.66	TRANSLATION
Q9D8N0	Eef1g	6.69	TRANSLATION
O70251	Eef1b	6.38	TRANSLATION
Q8BFR5	Tufm	1.96	TRANSLATION

Table S3: Manual assessment of key cellular machinery for Essential expression-restricted patterns of distant metastasis-free survival (DMFS) using GOBO dataset KM analysis.

Molecular Complex / Process	OFFICAL GENE SYMBOL	Essential Expression-Restricted	Patient groups
SWI/SNF BAF	SMARCD1	Yes	ER pos, Basal, ER pos LN neg, ER pos Her2enr, ER pos grade3
	ACTB		
	ACTL6B		
	ARID1A	Trending	Her2enr
	PBRM1		
	BCL7A	Yes	ER pos Normal like, ER pos grade 3
	BCL7B		
	BCL7C	Yes	Normal Like, ER pos Normal like
	BCL11A		
	BCL11B	Yes	Basal, grade3,
	BRD7	Trending	Lum B
	BRD9	Yes	ER pos, ER pos Lum A, ER pos LN neg, LN neg, Basal (trending)
	SMARCA4	Yes	ER pos Normal like
	CREST	Yes	LumA, ER pos LumA, ER pos her2enr (trending)
	EIF5B		
	GLTSCR1		
	SMARCB1		
	SMARCC1		
	SMARCD2		
	SMARCD3		
	SMARCE1		
	SMARCC2	Trending	ERBB2
	SMARCA2		
	PHF10		
	DPF1		
	DPF3		
	DPF2		
	All factors as a signature	Yes	Luminal A, ER pos, ER pos Luminal A, ER pos LN neg
6 out of 26 (23%) factors in this complex are essential expression-restricted metastasis modifiers			
ISWI (Imitation SWItch)	SMARCA5		
	BAZ1A	Yes	Luminal A
	CHRAC1		
	POLE3		
	MYO1C	Yes	All tumors tumors, ERBB2 (trending), Her2enr, LN neg, untreated
	SF3B1		
	MYBBP1A		
	DDYes21		
	RSF1		
	RBBP7		
	RBBP4		
	CECR2		
	ZMPSTE24		
	SMARCA1		
	BPTF		
	DEK		
	ERCC6		
	BAZ2A	Yes	Her2enr
	BAZ1B	Yes	Basal
	All factors as a signature		
4 out of 17 (23.5%) factors in this complex are essential expression-restricted metastasis modifiers			
CHD (Chromodomain, Helicase, DNA binding)	CHD1		
	CHD2	Trending	ER pos untreated (trending)
	CHD3	Yes	ER pos, ER pos LN neg, ER pos Grade 3, ER pos Untreated
	CHD4	Yes	ER neg
	CHD5	Yes	Grade 1, ER grade 1
	CHD6		
	CHD7		
	CHD8	Trending	ER pos ERBB2
	CHD9		
	HDAC1		
	HDAC2		
	MTA1		
	MTA2	Yes	ER pos, ER pos LN neg
	MTA3		
	GATAD2 A		
	GATAD2 B		
	MBD2		
	MBD3	Yes	ER pos Luminal B
	All factors as a signature	Trending	ER pos Untreated
5 out of 15 (33.3%) factors in this complex are essential expression-restricted metastasis modifiers			

Molecular Complex / Process	OFFICAL GENE SYMBOL	Essential Expression-Restricted	Patient groups
INo80 (INositol requiring 80)	INO80		
	RUVBL1		
	RUVBL2		
	MCRS1	Yes	Normal like tumors
	TFPT	Yes	Grade 2
	ACTL6A		
	YY1		
	INO80C		
	INO80B		
	UCHL5	Yes	LN neg, grade 3, Untreated,
	NFRKB		
	INO80E		
	MEAF6		
	MORF4L1	Yes	ER pos LN neg,
	MORF4L2	Trending	ER pos Her2enr
	MRGBP		
	SRCAP		
	EP400		
	YEATS4		
	EAF2	Yes	ER pos Her2enr
	YL-1		
	ARP6		
	ZnF-HIT1		
	TRRAP		
	TRIP60		
	BRD8	Trending	ER neg
	EPC1		
	EPC2		
	ING3		
	All factors as a signature	Trending and Yes	Luminal B, TAM, ER pos TAM
**5 out of 21 (23.8%) factors in this complex are essential expression-restricted metastasis modifiers**			
Mediator complex	MED1		
	MED4	Yes	ER neg, Her2enr,
	MED6		
	MED7		
	MED8		
	MED9		
	MED10		
	MED11		
	MED12		
	MED12L		
	MED13		
	MED14		
	MED15		
	MED16		
	MED17		
	MED18	Yes and Trending	ER pos LN neg, ER pos Her2enr
	MED19		
	MED20		
	MED21		
	MED22		
	MED23	Yes	Luminal A, ER pos Luminal A
	MED24	Yes	ER pos Luminal A,
	MED25		
	MED26		
	MED27	Yes	ER pos LN neg
	MED28	Yes	PAM50 Luminal B
	MED29		
	MED30		
	MED31		
	CCNC		
	CDK8	Yes	ER pos grade 3
	All factors as a signature	Trending	Luminal B, LN pos
**7 out of 23 (30%) factors in this complex are essential expression-restricted metastasis modifiers**			
Glycolysis gene set (PANTHER Pathways)	ALDOA		
	BPGM	Yes	Grade 1, ER pos ERBB2
	ENo1		
	ENo2		
	GAPDH	Yes	ER pos LN pos
	GPI		
	HK1		
	HK2		
	HKDC1	Yes	ERBB2, Her2enr, ER pos ERBB2, ER pos grade 3
	PFKL	Yes	ER Basal
	PFKM	Yes	Basal
	PGAM1		
	PGAM2		
	PGAM4		
	PGK1		
	PKLR		
	PKM2		
	TPI1		
	All factors as a signature		
**5 of 12 genes (41.66%) factors in this pathway are essential expression-restricted metastasis modifiers**			

Molecular Complex / Process	OFFICAL GENE SYMBOL	Essential Expression-Restricted	Patient groups
Citric Acid Cycle	ACO2		
	CS		
	FH		
	MDH1		
	OGDH	Yes	ER neg , Grade 3
	PDHA1		
	PDHA2	Yes	Her2enr, ER pos Her2enr
	SDHC	Yes	ER pos Luminal A,
	SUCLG1		
	All factors as a signature	Yes	Normal Like, LN neg, grade 3
3 of 9 (33.3%) factors in this pathway are essential expression-restricted metastasis modifiers			
mRNA Decay	CNOT1	Yes	ER pos Grade 3
	CNOT2		
	CNOT3		
	CNOT4		
	CNOT6		
	CNOT6L		
	CNOT7		
	CNOT8		
	RQCD1	Yes	LN neg, Untreated,
	CNOT10		
	CNOT11		
	All factors as a signature	Yes	Luminal A, Normal like
2 of 8 (25%) factors in this pathway essential expression-restricted metastasis modifiers			
Nonsense-mediated decay (NMD)	UPF1		
	UPF2		
	UPF3A		
	UPF3B	Yes	Luminal A, ER pos luminal A,
	MAGOH	Yes	Luminal A
	CASC3	Yes	TAM, ER pos Grade 3, ER pos TAM
	All factors as a signature	Yes	Luminal A
3 of 6 (50%) factors in this pathway essential expression-restricted metastasis modifiers			
SURF complex	SMG1	Yes	Luminal A
	UPF1		
	ETF1		
	ERF3		
	SMG7	Trending and Yes	Basal, Her2 Enriched
	SMG5		
	PP2A		
	SLBP	Yes	ER neg, basal
	UPF2		
	UPF3A		
	All factors as a signature	Yes	ER pos Luminal B
2 of 8 (25%) factors in this pathway essential expression-restricted metastasis modifiers			
Endoribonucleolytic decay			
	ATP2C1		
	ERN1	Yes	LN neg, ER pos LN neg, ER pos Untreated
	ABCC1		
	EIF2C2		
	TIA1		
	XBP1		
	All factors as a signature	Yes	Basal
1 out of 6 (22%) factors in this pathway are essential expression-restricted metastasis modifiers			
Deadenylation-independent decapping			
	RPS28	Yes	ER neg
	AMBP		
	EDC3		
	ACE	Yes	ERBB2, ER neg, Her2 enriched
	DCP2		
	All factors as a signature		
2 out of 5 (40%) factors in this pathway are essential expression-restricted metastasis modifiers			

Molecular Complex / Process	OFFICAL GENE SYMBOL	Essential Expression-Restricted	Patient groups
**Decapping and 5'→ 3' decay.**			
	ACE	Yes	ERBB2, ER neg, Her2 enriched
	DCP2		
	EDC4	Yes	Luminal A
	LSM14A		
	DHH1		
	EDC3		
	PAT1		
	All factors as a signature		
**2 out of 5 (40%) factors in this pathway are essential expression-restricted metastasis modifiers**			
**Deadenylation**			
	PAN2	Yes	All tumors, basal, ER pos, LN neg, ER pos LN neg, TAM
	CNoT8		
	CCR4	Yes	ER pos Luminal A
	PARN		
	EXOSC10		
	All factors as a signature	Yes	ER pos Luminal A, ER pos Luminal B
**2 out of 5 (40%) factors in this pathway are essential expression-restricted metastasis modifiers**			
**RNA Exosome**			
	EXOSC1		
	EXOSC2		
	EXOSC3		
	EXOSC4	Yes	All tumors tumors, her2enriched, LN neg, grade 1, ER pos,
	EXOSC5	Yes	Normal-like
	EXOSC6		
	EXOSC7		
	EXOSC8		
	EXOSC9		
	EXOSC10		
	All factors as a signature	Yes	Her2 enriched
**2 out of 9 (22%) factors are essential expression-restricted metastasis modifiers**			
**Summary**			
**Total genes assessed:**	**175**		
**Essential Expression-restricted:**	**51**		
**Percentage:**	**29%**		

Table S4 : qRT-PCR and PCR primer sequences.

Supplemental Table 2: qRT-PCR primer sequences		
Primer name A-O		primer sequence 5' - 3'
Adgrg6 f		GTAGGAGAGGACAGGATGAA
Adgrg6 r		CAGGCTGACGTTATTGGTAG
Apex2 f		CGAGCTGGATGCTGATATTG
Apex2 r		CGGCTGAAGCTGAAATAGG
Cpsf4 f		GAGAGGACTGTGCAAGAAAG
Cpsf4 r		TGGATTCAGGGTCGATGT
Dusp19 f		ATGTGCCTGAAACCAATATCC
Dusp19 r		CCTGGAAACACCTGCATTAC
E2f2 f		AGCACCTGACCGAAGATA
E2f2 r		TCACTGTCTGCTCCTTGA
Hirip3 f		GGAAGGAAAGCCAGACTTTAT
Hirip3 r		GATGGTGAAGAGCTGGATTC
Htra3 f		CGATGTGGTGGAGAAGATTG
Htra3 r		GCGTTGGTGACGATCAAA
Ints7 f		CAGAAGTGAAGGCGGTAATC
Ints7 r		CGTCAGCAAACTCTGGTAAA
Lynx1 f		ATGGCCACCTACTGTATGA
Lynx1 r		CCATCGTACACGGTTTCAA
Nanos1 f		CTTGAGCAATCAAGGTGGGT
Nanos1 r		CCGATGGTCCAATTCTTTGT
Ociad2 f		CACTCATGGGAACCAAGAAA
Ociad2 r		CTCTGTGGATGTGCAGTTT

r reverse primer
f forward primer

Supplemental Table 2: qRT-PCR primer sequences		
Primer name P-T		primer sequence 5' - 3'
Pcyt1b f		GCTGGTTCTGATGACGTTTA
Pcyt1b r		CTGGTGATGATGTCTGATGTT
Perp f		GCTTTGGTGGAGGTGTTT
Perp r		GCAGATGCACAGGATGATAA
Ppib f		GGAGATGGCACAGGAGGAAAGAG
Ppib r		TGTGAGCCATTGGTGTCTTTGC
Prr9 f		GTCCACATCCTTCATTGAATCT
Prr9 r		ATGGTTGCTTGCACTGTT
Pum2 f		GCTCAGCAGCCACATATAG
Pum2 r		TGGAGGAGCAGCACTAATA
Pvrl1 f		GTGAAAGCCAGCTCAATCT
Pvrl1 r		GTCATCCTGTCCCTTCCT
Rfng f		GTGAAATTCTGGTTTGCTACTG
Rfng r		CTCTGCTGTGCTCATGAAA
Robo3 f		CTACACATTGCCAGCATACA
Robo3 r		GCTTCCTAAGCCAGCTATTC
Rtkn2 f		GAAAGACTCCGATCACTTCAG
Rtkn2 r		CACCATGTCCGTGTCAAA
S100a4 f		GAACAAGACAGAGCTCAAGG
S100a4 r		TGTCCAAGTTGCTCATCAC
Scarb1 f		GAGCATTCCTTGTTCCTAGAC
Scarb1 r		CGATGCCCTTGACAGATTT
Smarcd1 f		ATGAGGAAGCGGCTAGAT
Smarcd1 r		CGGATTGAACGTGTTAGAAATG
Tmem252 f		CTACCGCCAAGCAAATGA
Tmem252 r		TCAACATCCAGGCTCTCT
Tob2 f		GGAAGCTGGGAGAGAGAC
Tob2 r		ACAGCCCAAGAGCAGAG

Supplemental Data Legends

Supplementary Data 1: Nanos1-, Pum2-, and Cpsf4-responsive genes, DAVID analysis, and Ingenuity Pathway Analysis.
Sheet 1: NANOS1-responsive genes. Differential gene expression analysis compared to control.
Sheet 2: PUM2-responsive genes. Differential gene expression analysis compared to control.
Sheet 3: CPSF4-responsive genes. Differential gene expression analysis compared to control.
Sheet 4: Venn diagram analysis. 21 genes commonly altered in all knockdown lines compared to control.
Sheet 5: DAVID functional clustering analysis of genes listed in sheet 4.
Sheet 6: IPA analysis of differential gene expression in NANOS1 KD cells compared to control.
Sheet 7: IPA analysis of differential gene expression in PUM2 KD cells compared to control.
Sheet 8: IPA analysis of differential gene expression in CPSF4 KD cells compared to control.

Supplementary Data 2: SMARCD1-responsive genes in monolayer and 3D culture.
Sheet 1: Differential gene expression analysis for *Smarcd1* overexpression (OE) vs empty vector (EV) control cells grown in monolayer.
Sheet 2: Differential gene expression analysis for *Smarcd1* knockdown (KD) vs shScr control cells grown in monolayer.
Sheet 3: Venn diagram analysis of differentially expressed genes in *Smarcd1* OE vs EV cells and KD vs shScr cells grown in monolayer.
Sheet 4: Differential gene expression analysis for *Smarcd1* OE vs EV cells grown in 3D.
Sheet 5: Differential gene expression analysis for *Smarcd1* KD vs shScr cells grown in 3D.
Sheet 6: Venn diagram analysis of differentially expressed genes in *Smarcd1* OE vs EV cells and KD vs shScr cells grown in 3D.

Supplementary Data 3: ATAC-seq annotated peaks.
Sheet 1: Annotated regions of open chromatin in 6DT1 empty vector (EV) cells grown in monolayer.
Sheet 2: Annotated regions of open chromatin in 6DT1 shScramble (shScr) cells grown in monolayer.
Sheet 3: Annotated regions of open chromatin in 6DT1 *Smarcd1* overexpression (OE) cells grown in monolayer.
Sheet 4: Annotated regions of open chromatin in 6DT1 *Smarcd1* knockdown (KD) cells grown in monolayer.
Sheet 5: Annotated regions of open chromatin in 6DT1 EV cells grown in 3D (spheres).
Sheet 6: Annotated regions of open chromatin in 6DT1 shScr cells grown in 3D (spheres).
Sheet 7: Annotated regions of open chromatin in 6DT1 Smarcd1 OE cells grown in 3D (spheres).
Sheet 8: Annotated regions of open chromatin in 6DT1 Smarcd1 KD cells grown in 3D (spheres).

Supplementary Data 4: Unique motif enrichment in open chromatin.
Sheet 1: Venn diagram analysis identifying significantly enriched motifs unique to each cell line.
Sheet 2: Significantly enriched motifs within open chromatin of 6DT1 empty vector (EV) cells grown in monolayer.
Sheet 3: Significantly enriched motifs within open chromatin of 6DT1 shScramble (shScr) cells grown in monolayer.
Sheet 4: Significantly enriched motifs within open chromatin of 6DT1 *Smarcd1* overexpression (OE) cells grown in monolayer.
Sheet 5: Significantly enriched motifs within open chromatin of 6DT1 *Smarcd1* knockdown (KD) cells grown in monolayer.
Sheet 6: Significantly enriched motifs within open chromatin of 6DT1 EV cells grown in 3D (spheres).
Sheet 7: Significantly enriched motifs within open chromatin of 6DT1 shScr cells grown in 3D (spheres).
Sheet 8: Significantly enriched motifs within open chromatin of 6DT1 Smarcd1 OE cells grown in 3D (spheres).
Sheet 9: Significantly enriched motifs within open chromatin of 6DT1 Smarcd1 KD cells grown in 3D (spheres).

Supplementary Data 5: Differential splicing Ingenuity Pathway Analysis (IPA) of differentially spliced transcripts in *Smarcd1*-altered cells.
Sheet 1: Differential splicing analysis for *Smarcd1* overexpression (OE) vs empty vector (EV) cells grown in monolayer.
Sheet 2: Differential splicing analysis for *Smarcd1* knockdown (KD) vs shScramble (shScr) cells grown in monolayer.
Sheet 3: Venn diagram analysis of differentially spliced transcripts in *Smarcd1* OE vs EV cells and KD vs shScr grown in monolayer.
Sheet 4: Differential splicing analysis for *Smarcd1* OE vs EV cells grown in 3D.
Sheet 5: Differential splicing expression analysis for *Smarcd1* KD vs shScr cells grown in 3D.
Sheet 6: Venn diagram analysis of differentially spliced transcripts in *Smarcd1* OE vs EV cells and KD vs shScr grown in 3D.
Sheet 7: IPA of differentially spliced genes in *Smarcd1* overexpression (OE) cells compared to control grown in monolayer.
Sheet 8: IPA of differentially spliced genes in *Smarcd1* knockdown (KD) cells compared to control grown in monolayer.
Sheet 9: IPA of differentially spliced genes in *Smarcd1* OE cells compared to control cells grown in 3D (spheres).
Sheet 10: IPA of differentially spliced genes in *Smarcd1* KD cells compared to control cells grown in 3D (spheres).
Sheet 11: Venn diagram analysis for monolayer-specific pathways enriched in alternate splice variants, categorized by cellular process.
