# Supplementary material for: SMARCD1 is an essential expression-restricted metastasis modifier
Source: Commun Biol. 2024 Oct 10;7:1299. doi: 10.1038/s42003-024-07018-3 (PMC11467182; doi:10.1038/s42003-024-07018-3)
Supplement: Supplementary file 2 — Description of Additional Supplementary Materials [file 42003_2024_7018_MOESM2_ESM.pdf]

Description of Additional Supplementary Files

File name: Supplementary Data 1

Description: Sheet 1: NANOS1-responsive genes. Differential gene expression analysis compared to control.

Sheet 2: PUM2-responsive genes. Differential gene expression analysis compared to control.

Sheet 3: CPSF4-responsive genes. Differential gene expression analysis compared to control.

Sheet 4: Venn diagram analysis. 21 genes commonly altered in all knockdown lines compared

to control.

Sheet 5: DAVID functional clustering analysis of genes listed in sheet 4.

Sheet 6: IPA analysis of differential gene expression in NANOS1 KD cells compared to control.

Sheet 7: IPA analysis of differential gene expression in PUM2 KD cells compared to control.

Sheet 8: IPA analysis of differential gene expression in CPSF4 KD cells compared to control.

File name: Supplementary Data 2

Description: Sheet 1: Differential gene expression analysis for Smarcd1 overexpression (OE) vs empty vector (EV) control cells grown in monolayer. Sheet 2: Differential gene expression analysis for Smarcd1 knockdown (KD) vs shScr control cells grown in monolayer. Sheet 3: Venn diagram analysis of differentially expressed genes in Smarcd1 OE vs EV cells and KD vs shScr cells grown in monolayer. Sheet 4: Differential gene expression analysis for Smarcd1 OE vs EV cells grown in 3D. Sheet 5: Differential gene expression analysis for Smarcd1 KD vs shScr cells grown in 3D. Sheet 6: Venn diagram analysis of differentially expressed genes in Smarcd1 OE vs EV cells and KD vs shScr cells grown in 3D

File name: Supplementary Data 3

Description: Sheet 1: Annotated regions of open chromatin in 6DT1 empty vector (EV) cells grown in monolayer. Sheet 2: Annotated regions of open chromatin in 6DT1 shScramble (shScr) cells grown in monolayer. Sheet 3: Annotated regions of open chromatin in 6DT1 Smarcd1 overexpression (OE) cells grown in monolayer. Sheet 4: Annotated regions of open chromatin in 6DT1 Smarcd1 knockdown (KD) cells grown in monolayer. Sheet 5: Annotated regions of open chromatin in 6DT1 EV cells grown in 3D (spheres). Sheet 6: Annotated regions of open chromatin in 6DT1 shScr cells grown in 3D (spheres). Sheet 7: Annotated regions of open chromatin in 6DT1 Smarcd1 OE cells grown in 3D (spheres). Sheet 8: Annotated regions of open chromatin in 6DT1 Smarcd1 KD cells grown in 3D (spheres).

File name: Supplementary Data 4

Description: Sheet 1: Venn diagram analysis identifying significantly enriched motifs unique to each cell line. Sheet 2: Significantly enriched motifs within open chromatin of 6DT1

empty vector (EV) cells grown in monolayer. Sheet 3: Significantly enriched motifs within open chromatin of 6DT1 shScramble (shScr) cells grown in monolayer. Sheet 4: Significantly enriched motifs within open chromatin of 6DT1 Smarcd1 overexpression (OE) cells grown in monolayer. Sheet 5: Significantly enriched motifs within open chromatin of 6DT1 Smarcd1 knockdown (KD) cells grown in monolayer. Sheet 6: Significantly enriched motifs within open chromatin of 6DT1 EV cells grown in 3D (spheres). Sheet 7: Significantly enriched motifs within open chromatin of 6DT1 shScr cells grown in 3D (spheres). Sheet 8: Significantly enriched motifs within open chromatin of 6DT1 Smarcd1 OE cells grown in 3D (spheres). Sheet 9: Significantly enriched motifs within open chromatin of 6DT1 Smarcd1 KD cells grown in 3D (spheres).

File name: Supplementary Data 5

Description: Sheet 1: Differential splicing analysis for Smarcd1 overexpression (OE) vs empty vector (EV) cells grown in monolayer. Sheet 2: Differential splicing analysis for Smarcd1 knockdown (KD) vs shScramble (shScr) cells grown in monolayer. Sheet 3: Venn diagram analysis of differentially spliced transcripts in Smarcd1 OE vs EV cells and KD vs shScr grown in monolayer. Sheet 4: Differential splicing analysis for Smarcd1 OE vs EV cells grown in 3D. Sheet 5: Differential splicing expression analysis for Smarcd1 KD vs shScr cells grown in 3D. Sheet 6: Venn diagram analysis of differentially spliced transcripts in Smarcd1 OE vs EV cells and KD vs shScr grown in 3D. Sheet 7: IPA of differentially spliced genes in Smarcd1 overexpression (OE) cells compared to control grown in monolayer. Sheet 8: IPA of differentially spliced genes in Smarcd1 knockdown (KD) cells compared to control grown in monolayer. Sheet 9: IPA of differentially spliced genes in Smarcd1 OE cells compared to control cells grown in 3D (spheres). Sheet 10: IPA of differentially spliced genes in Smarcd1 KD cells compared to control cells grown in 3D (spheres). Sheet 11: Venn diagram analysis for monolayer-specific pathways enriched in alternate splice variants, categorized by cellular process
