# Supplementary material for: SMARCD1 is an essential expression-restricted metastasis modifier
Source: Commun Biol. 2024 Oct 10;7:1299. doi: 10.1038/s42003-024-07018-3 (PMC11467182; doi:10.1038/s42003-024-07018-3)
Supplement: Supplementary file 8 — Reporting Summary [file 42003_2024_7018_MOESM8_ESM.pdf]

nature portfolio

Corresponding author(s): Kent Hunter

Last updated by author(s): Feb 29, 2024

Reporting Summary

Nature Portfolio wishes to improve the reproducibility of the work that we publish. This form provides structure for consistency and transparency in reporting. For further information on Nature Portfolio policies, see our Editorial Policies and the Editorial Policy Checklist.

Statistics

For all statistical analyses, confirm that the following items are present in the figure legend, table legend, main text, or Methods section.

n/a	Confirmed	
☐	☒	The exact sample size (n) for each experimental group/condition, given as a discrete number and unit of measurement
☐	☒	A statement on whether measurements were taken from distinct samples or whether the same sample was measured repeatedly
☐	☒	The statistical test(s) used AND whether they are one- or two-sided<br>*Only common tests should be described solely by name; describe more complex techniques in the Methods section.*
☒	☐	A description of all covariates tested
☐	☒	A description of any assumptions or corrections, such as tests of normality and adjustment for multiple comparisons
☐	☒	A full description of the statistical parameters including central tendency (e.g. means) or other basic estimates (e.g. regression coefficient) AND variation (e.g. standard deviation) or associated estimates of uncertainty (e.g. confidence intervals)
☒	☐	For null hypothesis testing, the test statistic (e.g. F, t, r) with confidence intervals, effect sizes, degrees of freedom and P value noted<br>*Give P values as exact values whenever suitable.*
☒	☐	For Bayesian analysis, information on the choice of priors and Markov chain Monte Carlo settings
☒	☐	For hierarchical and complex designs, identification of the appropriate level for tests and full reporting of outcomes
☒	☐	Estimates of effect sizes (e.g. Cohen's d, Pearson's r), indicating how they were calculated

Our web collection on statistics for biologists contains articles on many of the points above.

Software and code

Policy information about availability of computer code

Data collection	does not apply
Data analysis	Sequencing data was analyzed for gene counts, differential analysis, pathway analysis, and motif analysis using Partek Flow (Illumina). in vivo data was analyzed using the statistical suit in Graph Pad Prism 9.

For manuscripts utilizing custom algorithms or software that are central to the research but not yet described in published literature, software must be made available to editors and reviewers. We strongly encourage code deposition in a community repository (e.g. GitHub). See the Nature Portfolio guidelines for submitting code & software for further information.

Data

Policy information about availability of data

All manuscripts must include a data availability statement. This statement should provide the following information, where applicable:

- Accession codes, unique identifiers, or web links for publicly available datasets
- A description of any restrictions on data availability
- For clinical datasets or third party data, please ensure that the statement adheres to our policy

RNA-seq and ATAC-seq data is currently being uploaded to the Gene Expression Omnibus (GEO) and accession numbers will be made available as soon as possible.

Research involving human participants, their data, or biological material

Policy information about studies with human participants or human data. See also policy information about sex, gender (identity/presentation), and sexual orientation and race, ethnicity and racism.

Reporting on sex and gender	Does not apply.
Reporting on race, ethnicity, or other socially relevant groupings	Does not apply.
Population characteristics	Does not apply.
Recruitment	Does not apply.
Ethics oversight	Does not apply.

Note that full information on the approval of the study protocol must also be provided in the manuscript.

Field-specific reporting

Please select the one below that is the best fit for your research. If you are not sure, read the appropriate sections before making your selection.

☒ Life sciences ☐ Behavioural & social sciences ☐ Ecological, evolutionary & environmental sciences

For a reference copy of the document with all sections, see nature.com/documents/nr-reporting-summary-flat.pdf

Life sciences study design

All studies must disclose on these points even when the disclosure is negative.

Sample size	in vivo sample size was determined power analysis. For metastasis assays, which are highly variable, we use 10 mice per group and performed the experiment twice for a total combined number of 20 mice per group.
Data exclusions	If mice died unexpectedly before our set 28-day endpoint their tissue was excluded from analysis.
Replication	All in vivo data reported here is compiled from 2 separate replicates. All molecular biology data reported here is the result of 3 experimental replicates.
Randomization	Mice were randomized into cages (5 mice per cage) upon receipt from the vendor, and therefore before use in any of our experiments.
Blinding	Before mice were euthanized for tissue collection and tumor assessment the groups were blinded by another lab member.

Reporting for specific materials, systems and methods

We require information from authors about some types of materials, experimental systems and methods used in many studies. Here, indicate whether each material, system or method listed is relevant to your study. If you are not sure if a list item applies to your research, read the appropriate section before selecting a response.

Materials & experimental systems

n/a	Involved in the study	
☐	☒	Antibodies
☐	☒	Eukaryotic cell lines
☒	☐	Palaeontology and archaeology
☐	☒	Animals and other organisms
☒	☐	Clinical data
☒	☐	Dual use research of concern
☒	☐	Plants

Methods

n/a	Involved in the study	
☒	☐	ChIP-seq
☒	☐	Flow cytometry
☒	☐	MRI-based neuroimaging

Antibodies

Antibodies used	mouse anti-CPSF4 (1:100; Santa Cruz), mouse anti-Pumilio2 (1:1,000; Abcam), mouse anti-SMARCD1 (1:1000; Bethyl A301-595A), rabbit anti-BICRA (1:1000; Abcam ab302712), rabbit anti-SMARCA4 (1:1000; Abcam ab110641), mouse anti-Actin (1:10,000; Abcam),

	mouse anti-Myc-tag (1:1000; Cell Signaling). Goat anti-rabbit (Santa Cruz) and goat-anti-mouse (GE Healthcare). SMARCD1 antibody (Bethyl A301-594A) .
Validation	The CPSF4 and Pumillio antibodies were validated by the manufactures and also by probing western blots with q-PCR-confirmed samples that were either control or knockdown for CPSF4 or Pumillio respectively. The SMARCD1 A301-595A and A301=594A antibodies were validated bby Bethyl laboratories and also by using lysates from cells with Smarcd1 over expression and shRNA knockdown. anti BICRA and SMARCA4 were validated for by the manufacturer Abcam. The secondary mouse and rabbit antibodies are validated by the manufacturers and are standards in our laboratory.

Eukaryotic cell lines

Policy information about cell lines and Sex and Gender in Research

Cell line source(s)	4T1 and 6DT1 cells were purchased from ATCC.
Authentication	Cell lines were authenticated by SNV analysis after exome sequencing, confirming the mouse strain from which these lines were derived as well as the 6DT1 Kras G12D driver mutation.
Mycoplasma contamination	These cells were not tested for mycoplasma.
Commonly misidentified lines (See ICLAC register)	no commonly mis-identified lines were used in this study.

Animals and other research organisms

Policy information about studies involving animals; ARRIVE guidelines recommended for reporting animal research, and Sex and Gender in Research

Laboratory animals	Thi study included the use of female Balb/c mice from Charles River as well as female 001800 - FVB/NJ mice from Jackson Laboratory.
Wild animals	This study did not involve wild animals.
Reporting on sex	This study did not report on sex.
Field-collected samples	This study did not involve any field-collected samples.
Ethics oversight	Ethical guidance and approval was granted for the use of mice in this study by the NCI Bethesda Animal Use and Care Committee.

Note that full information on the approval of the study protocol must also be provided in the manuscript.

Plants

Seed stocks	No plants were used in this study.
Novel plant genotypes	No plants were used in this study.
Authentication	No plants were used in this study.
